# 494. Real-world effectiveness of tixagevimab/cilgavimab for COVID-19 pre-exposure prophylaxis in solid organ transplant recipients during the Omicron variant period

**DOI:** 10.1093/ofid/ofad500.563

**Published:** 2023-11-27

**Authors:** Gun Rojanakit, Sasisopin Kiertiburanakul, Sarinya Boongird, Napan Sutharattanapong, Teerapat Yingchoncharoen, Abhasnee Sobhonslidsuk, Jackrapong Bruminhent

**Affiliations:** Faculty of Medicine Ramathibodi Hospital, Mahidol University, Bangkok, Krung Thep, Thailand; Faculty of Medicine Ramathibodi Hospital, Bangkok, Krung Thep, Thailand; Faculty of Medicine Ramathibodi Hospital, Mahidol University, Bangkok, Krung Thep, Thailand; Faculty of Medicine Ramathibodi Hospital, Mahidol University, Bangkok, Krung Thep, Thailand; Faculty of Medicine Ramathibodi Hospital, Mahidol University, Bangkok, Krung Thep, Thailand; Faculty of Medicine Ramathibodi Hospital, Mahidol University, Bangkok, Krung Thep, Thailand; Faculty of Medicine Ramathibodi Hospital, Mahidol University, Bangkok, Krung Thep, Thailand

## Abstract

**Background:**

Pre-exposure prophylaxis (PrEP) with a combination of long-acting monoclonal antibodies with tixagevimab/cilgavimab (T/C) have provided viral neutralizing activity against SARS-CoV-2 and protected solid organ transplant recipients (SOTRs) that produced poor immunogenicity after COVID-19 immunization. However, real-world data on the effectiveness and safety of T/C in SOTRs during the Omicron wave is limited.

**Methods:**

A prospective study at a single transplant center was conducted to evaluate the effectiveness and adverse events (AE) of T/C among SOTRs during the BA.2.75 Omicron lineages period (August 2022 to February 2023). T/C of 150/150 and 300/300 mg doses were provided from August 2022 to December 2022 and after, respectively.

**Results:**

A total of 39 SOTRs comprising kidney (n= 29), liver (n=2), and heart (n=8) transplant recipients were included in the study. Thirty-three (85%) and 6 (15%) SOTRs received 150/150 and 300/300 mg of T/C, respectively. The median (IQR) age was 52 (35-62) years. Twenty-two (56%) had a prior history of COVID-19. The median (IQR) number of COVID-19 vaccine was 4 (3-4) doses. During the median follow-up of 6.35 (5-8.33) months, COVID-19 developed in 5 (12.8%) out of 39 SOTRs with a median (IQR) time of 73 (36-115) days from receiving T/C. None of those who received a 300/300 mg dose developed COVID-19. There was no severe COVID-19 or death observed. Five (12.8%) SOTRs reported mild and reversible AEs with pain at injection (n=2), fever (n=3), and muscle aches (n=1). There was no allograft rejection reported.

**Conclusion:**

Breakthrough SARS-CoV-2 infection remains among fully vaccinated SOTRs receiving T/C as PrEP and subjects to concurrent circulating strain in the community. However, the disease severity appears mild, and the short-term safety profile seems tolerable.

**Disclosures:**

**All Authors**: No reported disclosures

